# Optimization: In-Depth Examination and Proposition

**DOI:** 10.3389/fpsyg.2019.01398

**Published:** 2019-06-19

**Authors:** Huy Phuong Phan, Bing Hiong Ngu, Alexander Seeshing Yeung

**Affiliations:** ^1^ School of Education, Faculty of Humanities, Arts, Social Sciences and Education, University of New England, Armidale, NSW, Australia; ^2^ Institute for Positive Psychology and Education, Australian Catholic University, Sydney, NSW, Australia

**Keywords:** optimal functioning, optimization, index of optimization, energization, subjective well-being, positive psychology, cognitive load theory, optimizing effect

## Abstract

One notable concept that is of interest is a *person’s state of optimal functioning*. Achieving optimal functioning (e.g., subjective well-being at school), aside from personal autonomy, requires some form of “optimization.” *Optimization*, we argue, is more than just an “enhancement,” a “predictive effect,” and/or a “causal flow” between an independent variable (IV) and a dependent variable (DV). We note from existing literature that optimization has often been referred to without a clear, definitive explanation of what this term actually entails. At the same time, we acknowledge that unlike other areas of development (e.g., engagement), no theoretical article is available to explain the concept of optimization. This article considers a number of theoretical tenets for advancement: (1) the tenet of *three major criteria* that could assist in the explanation, assessment, and measurement of optimization, (2) the tenet of the *development of a methodological conceptualization* that could measure and assess optimization, and (3) the tenet of the “quantification” of optimization, and in particular, a proposed *index of optimization* and a corresponding scientific notation of “*γ*”, which we coin as an “optimizing effect.” Overall, we contend that this examination is insightful and holistic, seeking clarity into an important topical theme in psychology.

## Introduction

One notable line of research in psychology that has recently received considerable interest is the *operational nature of optimal functioning*. Optimal functioning, which may be in physical, cognitive, emotional, and/or social terms, emphasizes the importance of a person’s *inner strength, state of resilience, virtue*, and the *maximization in capability* (Source: Applied Psychology: Health and Well-being). Optimal functioning reflects the paradigm of *positive psychology* (Seligman and Csikszentmihalyi, 2000; Seligman, 2010), and may in the context of academia, involve the experience of mastery, and/or the achievement of an exceptional academic result. Optimal functioning in a nonacademic arena, likewise, may indicate a football player’s exceptional achievement to score 50 goals in one season, for example. This theoretical concept of optimal functioning is in direct contrast to personal experiences of stagnation and pessimism, highlighting weakness, sub-optimal performance, and minimal potential. The concept of optimal functioning therefore takes a positive perspective. However, what optimal functioning constitutes and how optimization of human functioning operates are not clearly defined and understood. The aim of this article is to conduct an in-depth examination of the theories related to the concept of optimization and to propose future directions for research advancement.

## Understanding Optimization

Relating to the concept of optimal functioning is a question that we, as researchers, have made concerted attempts to address: *how does a person reach an optimal state of functioning?* This important question has led to our numerous empirical and conceptual undertakings, which specifically focus on the complexity of the operational mechanism of optimal functioning. What causes an exceptional state of functioning? What actually occurs as a state of functioning improves from one level to that of another level? How does the cause of optimal functioning associate with a level of optimal functioning? These three major questions have, to date, formed the central premise of existing research inquiries and our own contributions. Understanding this complexity of optimal functioning (e.g., how a person reaches a state of optimal cognitive functioning) is innovative, especially in terms of educational and social practices for implementation. From the context of successful schooling, for example, we could capitalize on this line of research development and design appropriate educational programs and/or pedagogical strategies, which may closely align with the optimization of students’ learning experiences.

The study of the processes of optimal functioning, from our point of view, is emerging and has received moderate attention. We recognize there are some prominent theoretical tenets that have, likewise, considered the improvement of cognitive functioning. For example, Vygotsky’s (1978, 1981) *sociocultural theory of cognitive development* stipulates the potent impact of the contextual environment to shape a person’s cognitive development. Psychological tools and cultural artifacts, such as mathematical symbols and notations may mediate a student’s progress in his/her understanding of problem solving. In particular, Vygotsky (1978) makes reference to an important term, coined as the “zone of proximal development,” which depicts the difference between what a person can do without help and what he/she can do with help (e.g., scaffolding). Piaget’s (1963) *theory of personal constructivism*, somewhat different from Vygotsky’s (1978) theory, contends that cognitive growth arises from a person’s experience resolution of disequilibrium *via* means of adaptation. In school contexts, according to Piaget’s (1963) theory, effective learning occurs when a child experiences a mental state of cognitive conflict. Learning outcomes that do not stimulate intellectual challenges or “flow” (Csíkszentmihályi, 1990; Seligman and Csikszentmihalyi, 2000) are more likely, in this analysis, to limit enriched cognitive experiences.

In sum then, our brief introduction contends that an optimal state of functioning indicates personal growth, improvement, and exceptional performance. Achieving this optimal state of functioning requires some form of scaffolding from the external world. Notwithstanding existing theoretical contributions (Piaget, 1963; Vygotsky, 1978), one element that has gone amiss is an in-depth examination of the actual operation involved in the achievement of optimal functioning. This operation, from our point of view and proposition, is known as the process of “optimization” (Phan et al., 2019a, b). The term optimization, extensively used in the academic literature (e.g., Freund and Baltes, 1998; Fraillon, 2004; Ziegelmann and Lippke, 2007) is inconsistently explained, and has not been adequately addressed. The true nature of optimization, we argue, is relatively unknown at present in terms of analysis and understanding. What actually occurs during the process of optimization? How does the process of optimization explain a person’s optimal best practice? Can the process of optimization be “quantified” and be represented by a scientific notation? These questions indicate the totality of our understanding of optimal functioning.

## Optimal Functioning: An Introduction

*Optimal functioning* is a perceived positive theoretical concept that emphasizes the importance of improved competence, personal best or exceptionality, and a strong sense of motivation and resilience. Optimal functioning situated within the context of academia is also analogously termed as *optimal best practice* (Phan et al., 2016, 2018a) and *personal best* (Martin, 2006, 2011). An analysis of the literature indicates that, likewise, educators and researchers have often referred to the notion of an “optimal condition” for effective learning and enriched schooling experiences. An educator, for example, may consider strategies and/or programs that could stimulate and foster a positive social climate for learning, which in turn could instill a perceived sense of school belonging for students (Goodenow, 1993; Goodenow and Grady, 1993).

Optimal functioning is a central feat of human agency and may apply to different complex contexts in life (Straszewski and Siegel, 2018; Wiese et al., 2018). Optimal functioning, in this case, may consist of different facets – for example, optimal physical functioning, optimal cognitive functioning, optimal emotional functioning, etc. In the areas of health and subjective well-being, researchers have, for example, explored the concept of optimal subjective well-being (Fraillon, 2004; ACU and Erebus International, 2008). This research inquiry, indeed, has led to the propositions of a number of definitions and views about the nature of optimal functioning. The literature review published by the Australian Catholic University (ACU) in 2004 specifically elucidated the essence of optimal functioning, which the researchers expressed their understanding – “maximizing one’s potential” (Dunn, 1961; Ryff, 1995), “pursuit of excellence in physical, mental, emotional, and spiritual realm” (Ardell, 1982), “an active process of fulfillment” (Hettler, 1984), “living and working effectively” (Corbin, 1997), “living fully in the natural community” (Witmer and Sweeney, 1998), “resilience and successful community participation” (Weisner, 1998), “holistic, positive emotions” (Stewart-Brown, 2000), “positive emotions, life satisfaction, and absence of negative emotions” (Diener and Biswas-Diener, 2002), “positive feelings and positive psychosocial functioning” (Keyes, 2002), “resilience, satisfaction, and maximizing one’s potential” (Bornstein et al., 2003), “positive feelings and life satisfaction” (Headey and Wooden, 2004), “positive state and satisfaction of needs” (Prilleltensky and Prilleltensky, 2006), and “resilience and maximizing one’s potential” (WHO, 2007).

From this theoretical overview (ACU and Erebus International, 2008), a person’s achievement of optimal functioning indicates numerous attributes that are positive – for example, self-fulfillment and inner satisfaction, exceptional accomplishment, and enrichment and personal growth. Depending on the nature of the context, a person may experience different types of attributes when he/she achieves optimal functioning. From an educational perspective, optimal functioning in an academic subject may reflect different learning experiences: a student’s ability to continuously perform and achieve outstanding results in Year-8 mathematics and receiving an “A” grade at the end of the school term (Phan et al., 2017), or a student’s seeking of mastery to know the different pedagogical approaches that could enable in-depth understanding of a topical theme (i.e., ability to solve challenging transfer percentage problems) (Ngu et al., 2018). At the same time, aside from mastery and performance-based accomplishments, optimal functioning may indicate a student’s heightened state of motivation (e.g., intrinsic) to persist with his/her studies (Church et al., 2001; Elliot and Murayama, 2008). From a noneducational point of view, likewise, optimal functioning on a daily basis may indicate a person’s positive outlook about life, and his/her strong state of personal resolute and resilience to combat health-related matters. Low optimal functioning, in this case, may result in feelings of pessimism and helplessness, and a belief that existing health issues are not worth combatting.

Overall then, from the aforementioned description, we contend that optimal functioning is an important element of a person’s development. Optimal functioning, in its simplistic summation, is concerned with an individual state of “change” that a person experiences for the better. Job satisfaction, combatting health, a positive outlook of life, personal best in sports performance, and successful schooling are some examples of a person’s positive experience of optimal functioning. Of relevance and significance in this discussion, which we next discuss, is an in-depth analysis and understanding of how an optimal level of functioning is accomplished. For example, within the context of academia, we want to consider in-class pedagogical strategies, school-based educational programs, and/or the use of intellectual capitals to enhance and optimize students’ cognitive functioning. This feat concerning the *nature of achievement* of optimal functioning has not been adequately addressed. We do not have clear evidence at present, both conceptually and empirically, to explain how a state of optimal best is ascertained. What are the underlying processes, which may govern our drive to achieve a state of exceptionality?

Our proposition of a detailed conceptualization of optimization, which may explain the intricate processes of achievement of optimal functioning, draws from existing theorizations (e.g., Csíkszentmihályi, 1990; Fraillon, 2004; Phan et al., 2017) and empirical research findings (Martin, 2011; Liem et al., 2012; Phan et al., 2018a,b,c). Optimization, as we conceptualize, is not an outcome or a relationship, but rather depicts an underlying process that in turn “optimizes” an entity in question (e.g., a person’s academic learning experience in a subject matter). In addition, we have also recently considered a related theoretical matter, namely, the conceptualization and development of appropriate methodologies that could enable the assessment and validation of optimization. This research-based discourse is innovative as emphasis is placed on researchers’ theoretical contributions to the study of a conceptualized inquiry.

## The Operational Nature of Optimization

What is optimization? In the preceding sections, we mentioned that optimization is an intricate process that closely aligns with the achievement of optimal functioning. An examination of the literature indicates that researchers have extensively used the term optimization in their researches (e.g., Freund and Baltes, 1998; Fraillon, 2004; Ziegelmann and Lippke, 2007). We contend there is ambiguity as to what optimization actually entails as a process. From a generic, simple point of view, optimization may be perceived as a “vehicle” that operates to maximize a person’s state of functioning from T_1_ to T_2_. In recent years, researchers in the areas of *subjective well-being* (Fredrickson, 2000; Keyes et al., 2002) and *healthcare and aging for senior citizens* (Freund and Baltes, 1998; Ziegelmann and Lippke, 2007) have made extensive reference to the concept of optimization. For example, in relation to healthcare for senior citizens, a number of researchers have theorized that optimization serves as a process of engagement in goal-directed actions and means to pursue and maintain personally relevant goals (e.g., a goal of adopting and maintaining a physically active lifestyle). In relation to the study of positive psychology, likewise, Noble and McGrath (2008) proposed a *Positive Educational Practices* (PEPs) *Framework* that focuses on five specific foundations of well-being, namely: (1) social and emotional competency, (2) positive emotions, (3) positive relationships, (4) engagement through strengths, and (5) a sense of meaning and purpose. This framework, according to the authors, provides guidance to educators, school administrators, and researchers in the optimization of positive educational initiatives. The PEPs Framework, in this case, facilitates and encourages students to find a sense of meaning at school, and a purpose in life. In a similar vein, Seligman’s (e.g., Seligman and Csikszentmihalyi, 2000; Seligman, 2010, 2011) work on the *PERMA Framework* has also acknowledged the importance of happiness, resilience, and personal growth. One central aspect of human endeavor encompasses an inner desire and striving for one to lead and live a meaningful and enriching life.

Other researchers, similarly, have explored other comparable concepts that we believe reflect the relatedness to the process of optimization. Diener (e.g., Diener et al., 2009, 2010) and other colleagues (e.g., Keyes, 2002; Huppert and So, 2013) have explored the concept of *flourishing*, which is defined as a person’s experience that life is going well. In a similar vein, a research focus on the proactivity and enrichment of life has led to the propositions of theoretical constructs such as *thriving* (Su et al., 2014; Wiese et al., 2018), defined as “a state of positive functioning at its fullest range” (Su et al., 2014), and *personal striving* (Phan and Ngu, 2015; Phan et al., 2018a,b,c), defined as “a person’s effort attempt to seek out realistic and/or ambitious endeavor for accomplishment” (Phan et al., 2018a,b,c). Flourishing, thriving, and personal striving are in accord with the paradigm of positive psychology (Seligman and Csikszentmihalyi, 2000; Seligman, 2010), and place emphasis on a person’s seeking to achieve optimal endeavors.

Understanding the true mechanism of optimization, theoretically and/or empirically, is relatively unknown at this stage. This consideration, in particular, depicts the finer detail of the “steps” involved in the achievement of optimal functioning. A few researchers have, in this instance, provided comparable explanations of the operational nature of optimization. Fraillon’s (2004) discussion paper on the subject of student well-being, for example, described an interesting tenet – namely a person’s *actual best functioning* (ABF) and his/her subsequent *notional best functioning* (NBF). Optimization, for the author, is defined as the difference between ABF and NBF. From Fraillon’s (2004) brief account, Phan et al. (2017) presented an elaborated conceptualization of the relationship between two levels of functioning – *realistic best* (RB) and *optimal best* (OB). Importantly, the authors’ conceptualization proposes an important element coined as the “zone of optimization,” which is defined as the difference or range between RB and OB. The zone of optimization varies in the magnitude of the difference or range between the two levels of functioning. Moreover, as a point for consideration, the zone of optimization seeks to explain the “amount” of optimization that would be needed to help optimize the achievement of OB from RB.

**Figure 1:**
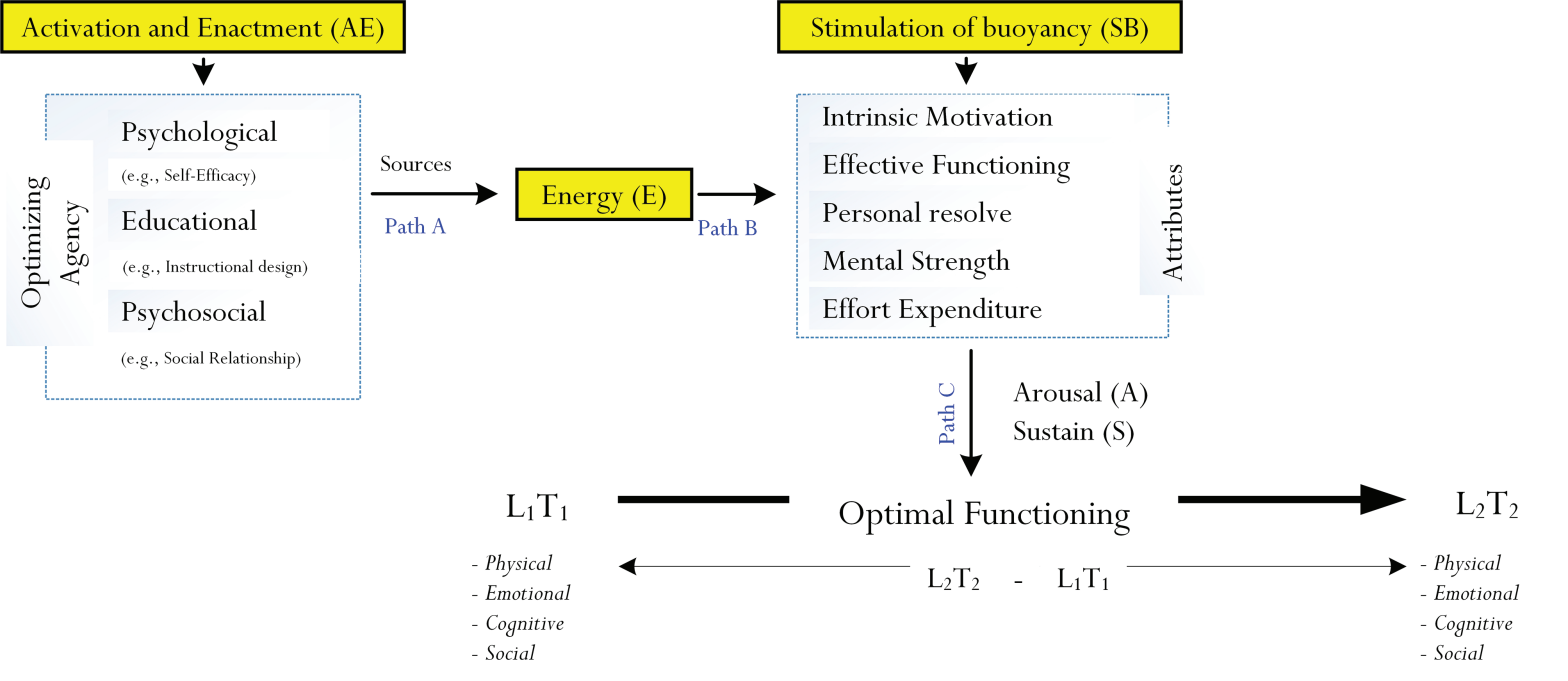
Proposition of the operational nature of the process of optimization.

Fraillon’s (2004) initial, but brief description of optimization and Phan et al.’s (2017) subsequent analysis both have provided theoretical grounding for further development. In our own recent research inquiries pertaining to the nature and scope of *mindfulness* (Phan et al., 2019a,b), we offered an expanded perspective and explanation of optimization. Our conceptualization, as shown in Figure 1, is more detailed and technical. In terms of different levels of functioning (e.g., RB: Phan et al., 2017), we argue that *time precedence* is an important element for incorporation – in other words, different levels of functioning cannot take place simultaneously.

From Figure 1, we propose that optimal functioning is the result of a progression from an existing level of functioning, denoted as L_1_, to a level that is more exceptional, denoted as L_2_. Mathematically, in this analysis, a person’s progression from L_1_ to L_2_ is denoted as Δ_(L_2_–L_1_)_. We argue for the inclusion of time difference because, as Fraillon (2004) and Phan et al. (2017) concur, L_1_ is indicative of a what person is capable of at present, whereas L_2_ is concerned with his/her maximum outcome. Being able to achieve L_2_ from L_1_ does not occur instantaneously, but rather requires an adequate timeframe for completion. Hence, from our conceptualization, we equate L_1_ to situate at T_1_ and L_2_ to situate at T_2_ – hence, overall, the achievement of optimal functioning may be defined as Δ_(L_2_T_2_–L_1_T_1_)_.

**Figure 2:**
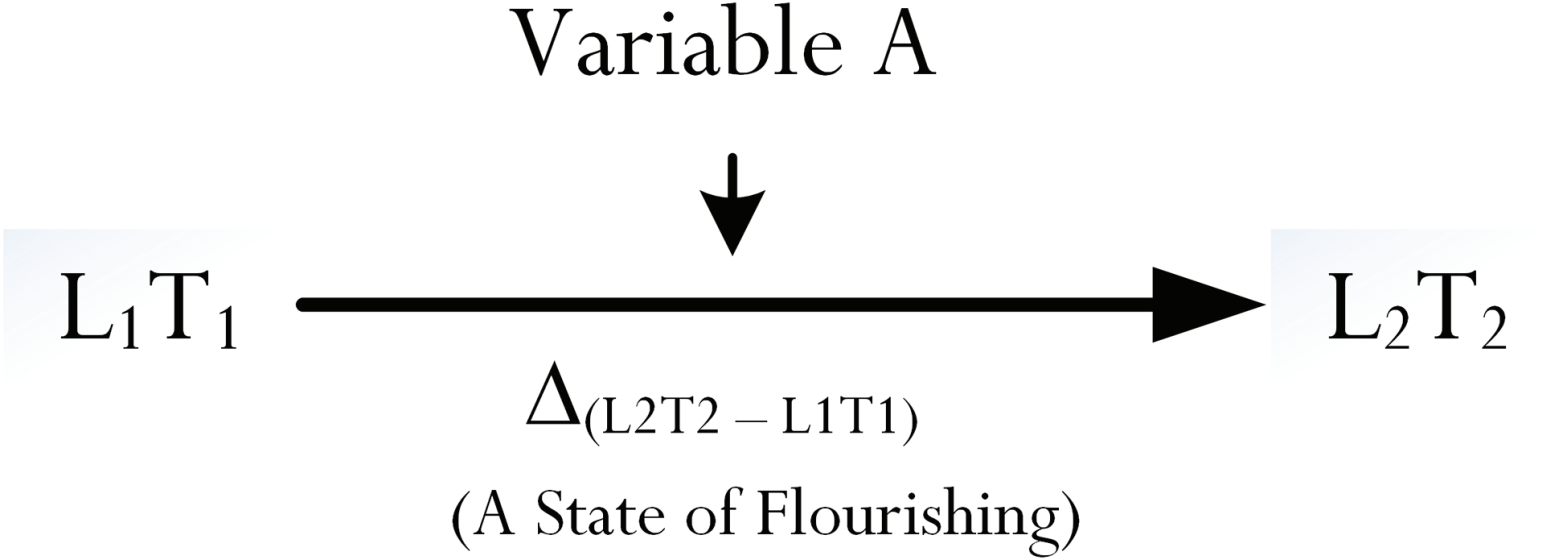
Simple methodological design of optimal functioning.

Methodologically, from a quantitative point of view (Tabachnick and Fidell, 2007), we may consider the assessment, measurement, and validation of Δ_(L_2_T_2_–L_1_T_1_)_. Social science’s research has used complex quantitative methodological designs to investigate associative and predictive effects of psychological and educational variables. Nonexperimentally, in this instance, we could consider the introduction of a variable A, which is then proposed to help “optimize” the improvement in score of L_1_ to L_2_ (Figure 2). Moreover, we expect to find that Δ_(L_2_T_2_–L_1_T_1_)_ would be positive in value. This proposition, in this case, stipulates an association between Variable *A* and Δ_(L_2_T_2_–L_1_T_1_)_.

Baron and Kenny’s (1986) seminal publication has resulted in extensive research development into the importance of mediating effects of variables (e.g., Grice et al., 2015; Kline, 2015; Tate, 2015; Trafimow, 2015). In this analysis, referring to our explanation, a central variable *A* could operate to mediate the effect of L_1_ at T_1_ onto L_2_ at T_2_ which can be tested in a series of path models: (1) *Test 1*: estimates a model in which only L_1_ predicts *L*_2_, (2) *Test 2*: estimates a model in which only Variable *A* predicts L_2_, (3) *Test 3*: estimates a model in which only L_1_ predicts Variable *A*, and (4) *Test 4*: assesses the reduction in the path from L_1_ to L_2_ with the introduction of Variable *A* as a mediator. Importantly though, in order to determine a true mediating effect and the potency of a mediator, we need to have evidence of *causal effects*, which in this case requires an experimental treatment or treatments, and the precedence of time difference.

Referring to Figure 2, and in tandem with Baron and Kenny’s (1986) criteria, it is poignant for us to consider the use of an intervention between T_1_ and T_2_. Referring to our previous discussion, Variable *A* could be considered as an “optimizing variable” between a determinant, L_1_, and an outcome, L_2_ – that is: L_1_ at T_1_ (determinant) → Variable *A* (optimizing variable) → L_2_ at T_2_ (outcome). In sum, from this introduction of a simple methodological design of optimal functioning, we propose three major criteria:

1. L_1_ as an informational source, which then serves as a *point of reference* for the achievement of L_2_.
2. The requirement of *timeframe* in order for a person to develop and experience an “increase” in optimal functioning (e.g., emotional functioning) – that is, the existence of multiple time points, which correspond with different levels of functioning – for example, L_1_ at T_1_, L_2_ at T_2_, etc.
3. The introduction of an *intervention*, which could operate as an “optimizing agent” in order to enhance and optimize L_1_ to L_2_.

Aside from a methodological account, we also need to consider the nature of Variable *A*. How does Variable *A* operate in order to facilitate an improvement of L_2_ from L_1_? The operational nature of Variable *A*, we contend, is intricate for its variation, which closely associates with the *complexity* of the Δ_(L_2_T_2_–L_1_T_1_)_. This proposition is similar to Phan et al.’s (2017) zone of optimization, whereby this “zone” differs and connotes a magnitude in strength for the process of optimization. What does this actually mean? For example, in relation to optimal health functioning, a person may require a substantial time period to combat an illness. An optimizing agent (i.e., Variable *A*) to improve the person’s health, in this case, may consist of an effective therapy, varying in intensity in accordance with the difference between L_1_ and L_2_. In a similar vein, a secondary school student wishing to achieve an optimal level of best practice in the topical theme of essay composition, based on his/her previous experiences, may require a lesser amount of time in terms of optimization. An optimizing agent to address Δ_(L_2_T_2_–L_1_T_1_)_ for writing composition may consist of an academic skills program that also vary in intensity.

### An In-Depth Analysis of the Operational Nature of Optimization

Variable *A*, as depicted in Figure 2, is proposed to operate as an optimizing agent, which then optimizes and enhances the achievement of L_1_ to L_2_. This proposition has been extensively detailed in Phan et al.’s (2017) theorization of optimization. From a methodological point of view, we could treat Variable *A* as a mediator between L_1_ and L_2_, and that there is a direct predictive path from L_1_ to L_2_. However, aside from its assessment and measurement, we contend that the totality and operational nature of Variable *A* is much more complex than it being viewed and treated as a mediator. Phan et al. (2017), in this case, proposed an underlying process encompassing this complexity, which comprises of two major sub-processes:

1. *Sub-process 1* concerns the “enactment” of different types of *psychological* (e.g., the positive impact of hope: Snyder, 2004), *educational* (e.g., an appropriate pedagogical practice: Ngu et al., 2014), and *psychosocial* (e.g., the complexity of the home environment: Daulta, 2008) *agencies*, which then initiate sub-process 2.
2. *Sub-process 2* involves the activation of the attributes of *persistence, effort expenditure*, and *effective functioning*, which then operate to optimize a state of functioning.

Both Fraillon’s (2004) consideration and Phan et al.’s (2017) theoretical model of optimization suggests that the process of optimization is more than just a directional association between sub-process 1 and sub-process 2. The “totality” of optimization, we propose, encompasses the stimulation and enrichment of experience of *vitality and buoyancy*. In this analysis, the enactment of optimization is likely to result in an enriched state of energy, strength, and liveliness, which would then enable a person to engage in proactive functioning. This complexity, we contend, is more accurately indicative of what actually occurs within the process of optimization. As shown in Figure 1, there are three pathways: Path A, Path B, and Path C. These paths tend to operate in a sequential manner, following these steps:

1. *Step 1*: This step, in line with Phan et al.’s (2017) theorization, is concerned with the activation and enactment (i.e., denoted as “AE”) of different psychological (e.g., the impact of hope: Snyder et al., 2000), educational (e.g., an appropriate instructional design: Ngu and Yeung, 2013), and/or psychosocial (e.g., the impact of teacher-student relationship: Roorda et al., 2011) agencies that then serve as sources of a person’s state of “energy” (we denote this as “E”). We argue that the activation and enactment of a particular agent (e.g., psychological agency) does not necessarily influence cognitive or motivational processes directly. Rather, the execution (i.e., activation and enactment) of an optimizing agent (e.g., psychological agent) serves to produce an experience of high “energy.” *Energization* is therefore an underlying sub-process of optimization, which in this case entails the experience and indication of vitality and buoyancy, assisting a person to stay focused on task.
  Vitality, from our conceptualization, forms a central element of the process of optimization. Personal experience of vitality (e.g., “I feel very energized at the moment”) is positive and enriching, predisposing a person to strife for the achievement of an optimal state of functioning. Importantly, of course, vitality is concerned with the observation and reporting of “stamina and liveliness” in cognition, behavior, and/or emotion, contrasting to a state of pessimism and procrastination, which correspondingly associate with a low level of energy. The selection, activation, and enactment of a specific optimizing agent (e.g., the use of hope as a psychological agent), in this case, depend upon the type of optimal functioning that a person is striving to achieve (e.g., optimal cognitive functioning in the area of mathematics).
2. *Step 2*: Personal experience of energization from Step 1 is postulated to stimulate the buoyancy of five comparable psychological attributes: *intrinsic motivation* (i.e., defined as a person’s intrinsic motive to persist a course of action – for example, learning Calculus), *personal resolve* (i.e., defined as a person’s internal state of decisiveness and resolute to strive for optimal functioning), *effective functioning* (i.e., defined as a person’s purposive state of organization, structured thoughts, and behavioral patterns and a deliberate intent to succeed), *mental strength* (i.e., defined as a person’s mindset of having the capacity to deal with obstacles, stressors, and pressure – for example, a tennis player is able to bounce back after losing two out of three games in competition tournament), and *effort expenditure* (i.e., a person’s conscious attempt to invest effort in order to achieve a particular outcome).
  Further to Fraillon’s (2004) brief description and Phan et al.’s (2017) subsequent conceptualization of optimization, we offer an expanded analysis where the sub-process of energization positively influences the operational nature of different types of psychological attributes (e.g., the stimulation of buoyancy of effort) that we perceive as being positive in nature. For example, one psychological attribute that we propose as being potent is a person’s internal mental strength to persevere, whereas another notable and related attribute is that of effort expenditure. Our recent correlational research, likewise, has attested to the direct and mediating effects of personal resolve and effective functioning (e.g., Phan et al., 2018a,b,c, 2019a).
3. *Step 3*: The stimulation of buoyancy of intrinsic motivation, personal resolve, effective functioning, mental strength, and effort expenditure *via* positive energy is postulated to *arouse* a person’s state of functioning at T_1_ at and sustain it to T_2_ (e.g., optimal cognitive functioning) (i.e., denoted as “AS”). For example, within the context of secondary schooling, the stimulation of buoyancy of intrinsic motivation may arouse a student’s interest in understanding Calculus, which could then help sustain a state of cognitive functioning. The student’s aroused state of cognitive functioning of mathematics learning, sustaining in progress from T_1_ to T_2_, may also involve the stimulation of buoyancy of effort expenditure, personal resolve, etc.
  A person’s aroused and sustained state of functioning within a particular context (e.g., academic learning in a subject matter) reflects the effectiveness of the stimulation of buoyancy of different types of psychological attributes. The effective stimulation is facilitated by an enriching state of energy, which arises from the activation and enactment of a relevant educational, psychological, and/or psychosocial agent. A low level of energy, in contrast, is likely to produce the inaction of different types of psychological attributes, resulting in sub-optimal functioning.

In summary, the pivotal components of optimization consist of the activation and enactment of psychological, educational, and psychosocial agencies, which then serve as sources of energy in order to stimulate the buoyancy of the five mentioned comparable attributes. Intrinsic motivation, personal resolve, effective functioning, mental strength, and effort expenditure in turn would individually, and/or in tandem, arouse and sustain a person’s progress in functioning from T_1_ to T_2_. In its simplistic term then, we can summarize the operational nature of optimization as follows: *AE* + *E* + *SB*.

Our methodological conceptualization of optimization, which we theoretically derive from previous inquiries (Fraillon, 2004; Phan et al., 2017), partially reflects Vygotsky’s (1978, 1981) *sociocultural theory of cognitive development* as well as other theories. For example, aligning to Vygotsky’s (1978, 1981) sociocultural theory of cognitive development, our conceptualization highlights three major facets: (1) extensive contributions from an external agent, especially in terms of the provision of opportunities of different types of agency for achievement of optimal functioning (e.g., a child’s exposure to different instructional designs/pedagogical practices from a teacher: Ngu et al., 2014), (2) the “internalization” of a particular agent and its “transformation” into a form of positive energy, and (3) the progress in a person’s state of functioning (e.g., cognitive functioning), consequently, as a result of external scaffolding. Other researchers, in contrast, have been less clear in their explanatory accounts and descriptions of optimal functioning and optimization-related entities.

## Methodological Development of Optimization

Our theoretical development of optimization has also led us to consider an important inquiry – namely, the development of what we coin as “methodological conceptualization,” which places an emphasis on the measurement, assessment, and evaluation of optimal functioning, and more importantly, the process of optimization. This methodological inquiry has theoretical, methodological, and empirical implications for consideration. From the perspective of quantitative methodology in the social sciences, there is acknowledgment that researchers may use both experimental and nonexperimental research designs to study associative patterns between variables (Bordens and Abbott, 2008; Gravetter and Forzano, 2009; Babbie, 2014). An important question for discussion then, is how do we measure, assess, and quantify the process of optimization?

### Assessing Optimal Functioning

From the preceding sections, the concept of optimal functioning reflects a number of analogous attributes, such as “personal best,” “maximization in capability,” “fullest potential,” and “exceptionality.” Our previous discussion has emphasized a reference point (e.g., T_1_) for benchmarking and comparison – this reference point may be denoted as L_1_T_1_, where L_1_ = initial level of functioning (e.g., cognitive functioning), T_1_ = time 1. For example, in the area of mathematics learning, we could consider a student’s *current* cognitive competence to solve *linear equations* with one unknown, *x* (e.g., solve for *x*: 5*x* – 11 = 4), as L_1_. This initial level of cognitive functioning (i.e., L_1_), known as actual functioning in Fraillon’s (2004) terms, or realistic achievement best in Phan et al.’s (2017), is postulated to act as a focal point for benchmarking. The student’s optimal level, denoted as L_2_ and benchmarked against L_1_, may consist of a competence to solve *quadratic equations* with one unknown, *x* [e.g., solve for *x*: (*x* – 5)^2^ = 20]. L_2_ (i.e., learning quadratic equations), compared to L_1_ (i.e., learning linear equations), is more advanced in terms of quality and cognitive complexity.

The achievement of optimal functioning from a current state of functioning, reflecting personal growth (i.e., “increase in a state of functioning”), may be defined as follows: ΔL_21_ = L_2_ T_2_ – L_1_ T_1_. From a quantitative point of view, we need to equate L_1_ and L_2_ with actual numerical values in order to determine what ΔL_21_ is. The “equating” of L_1_, L_2_, etc., with specific numerical values is subjective – that is, a student may equate L_1_ (i.e., knowing how to solve for *x*: 5*x* – 11 = 4) with an arbitrary value of 12 (e.g., out of 20), say, and L_2_ (i.e., knowing how to solve *x*: (*x* – 5)^2^ = 20) with an arbitrary value of 15 (e.g., out of 20), etc. Why do we want to quantify L_1_, L_2_, etc.? We contend that quantifying L_1_, L_2_, etc. with numerical values (e.g., 9, 10, 11, …, etc.) makes it relatively easy for researchers to rationalize the meaning of ΔL_21_, ΔL_32_, etc. In other words, quantitatively, an optimal level of functioning is more meaningful when it is denoted by a definitive numerical value.

Quantifying different levels of functioning with numerical values (e.g., “Provide an arbitrary score that you believe best describes your current level of emotional functioning”), of course, may pose a few problems for researchers, such as *inconsistency, subjective bias*, and *miscalibration*. A student’s inexperience in personal reflection, for example, may result in unintentional biased alignment of L_1_ (e.g., 3 out of 20), L_2_ (e.g., 12 out of 20), etc. when, in fact, this is not the case. Researchers focusing on students’ self-efficacy for academic learning (Bandura, 1986, 1997), likewise, have reported on the problem of underestimation and overestimation of judgments of perceived competence (Pajares and Kranzler, 1995; Pajares, 1996a,b). This problem of miscalibration of competence beliefs (e.g., underestimation), we contend, may arise from a student’s lack of focus, lack of concentration, and misunderstanding of instruction.

Aside from instructing a person to equate his/her level of an internal state of functioning with a corresponding numerical value, it is also possible to use Likert-scale measures and/or open-ended surveys. In the broad area of *subjective well-being*, for example, a number of researchers have developed different Likert-scale measures, such as the Comprehensive Inventory of Thriving (CIT) Scale (Su et al., 2014), the Flourishing Scale (Diener et al., 2010), and the Academic Striving Subscale (Phan et al., 2018a,b,c). The use of Likert-scale measures, administered to subjects on multiple occasions, is straightforward and may provide fruitful information about their current state of functioning and the potential of achieving optimal functioning. For example, consider a participant’s response to the Flourishing Scale (Diener et al., 2010) on two occasions, denoted as: Response-FT_1_ to Response-FT_2_. A positive change in scores from Response-FT_1_ and Response-FT_2_ (Δ_(Response-FT2 – Response-FT1)_ = +ve), in this analysis, would indicate an improvement in personal flourishing from T_1_ to T_2_. A negative difference (Δ_(Response-FT2 – Response-FT1)_ = −ve), in contrast, would suggest a decline in a person’s state of flourishing. It is possible, too, for us to explore and identify linear and/or nonlinear trajectories of a person’s subject well-being. The use of latent growth modeling (LGM) procedures, in particular, may also enable researchers to test for effects of extraneous influences on growth trajectories (Bollen and Curran, 2006; Hancock and Lawrence, 2006).

More recently, in an attempt to study the process of optimization (Fraillon, 2004; Phan et al., 2017), Phan et al. (2016) developed a Likert-scale questionnaire to measure and assess current level and optimal level of subjective academic well-being. The *Realistic Achievement Best Subscale* (e.g., “I am content with what I have accomplished so far for this subject”), according to the authors, explores a person’s actual functioning, whereas the *Optimal Achievement Best Subscale* (e.g., “I can achieve much more in this subject than I have indicated through my work so far”) reflects the person’s notional best functioning. The Optimal Outcome Questionnaire, as Phan et al. (2016) proposed, may serve as a diagnostic tool to assess students’ “profiles” of cognitive competence in their academic learning (Phan et al., 2018a,b,c). Furthermore, in their detailed theorization of optimization, Phan et al. (2016) postulate the forming of two subscale scores [i.e., the Realistic Achievement Best (RAB) Subscale and the Optimal Achievement Best (OAB) Subscale scores] that would assist in the assessment, measurement, and evaluation of the process of optimization. What is unclear though, from this consideration, is how we could use the RAB and OAB scores to measure and assess the operational nature of optimization.

From an educational perspective then, measuring and assessing a current level of cognitive functioning and an optimal level of cognitive functioning may involve the use of comparable quantitative methodologies, such as Likert-scale measures and cognitive competence tests (e.g., quiz). A robust methodological approach, in this case, may consist of an integration of three comparable measures: Likert-scale measures, the Optimal Outcome Questionnaire (Phan et al., 2016), and standardized testing (Phan et al., 2019a,b). This methodological conceptualization is depicted as follows:

**Figure:**
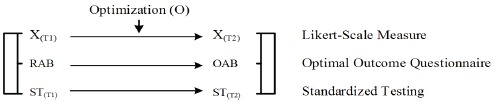


*Note*: the Likert-scale measure X = Comprehensive Inventory of Thriving (CIT) Scale (Su et al., 2014), the Flourishing Scale (Diener et al., 2010), the Academic Striving Subscale (Phan et al., 2018a,b,c), etc.

The above methodological conceptualization is insightful as it enables us to cross-validate the three comparable measures. The Optimal Outcome Questionnaire is administered to participants at a particular time point (Phan et al., 2016, 2017), and measures and assesses a person’s current level of functioning (i.e., the RAB Subscale) and his/her optimal level of functioning (i.e., the OAB Subscale). Of concern, from our viewpoint, is whether the OAB score actually indicates a person’s optimal best, or whether it is simply an indication of “miscalibrated” and potential optimal best (Phan et al., 2018a,b,c). On this basis, it would be appropriate to cross-validate the Optimal Outcome Questionnaire with another comparable Likert-scale measure (e.g., coined as “X”), administered to a participant on multiple occasions. Longitudinal research designs emphasize the importance of *time precedence*, stipulating the administration of the same Likert-scale measure on multiple occasions (Rogosa, 1979; MacCallum and Austin, 2000) – for example, a current time point, T_1_, and a future time point, T_2_. Hence, considering the Optimal Outcome Questionnaire and Likert-scale measure X, we propose a first iteration (*Iteration 1*) where there are two associations: (1) *r*_XT1-RAB_, which depicts the association between a Likert-scale measure X administered at T_1_ and the RAB Subscale, and (2) *r*_XT2-OAB_, which depicts the association between the same Likert-scale measure X administered at T_2_ and the OAB Subscale. In this analysis then, in terms of consistency and accuracy, we would expect similar rating scores for the RAB Subscale and the Likert-scale measure X at T_1_, and for the OAB Subscale and the same Likert-scale measure X at T_2_, respectively.

A scrutiny of the possibility of miscalibration is important (e.g., Pajares and Miller, 1994; Pajares and Kranzler, 1995; Pajares, 1996a,b) because miscalibration may result in either inflated (e.g., overconfidence of optimal level of cognitive functioning) or deflated (e.g., underconfidence of current level of cognitive functioning) responses. In a similar vein, the use of an identical Likert-scale measure on multiple occasions also poses problems such as identification of familiarity of items, and evidence of autocorrelated errors between items – for example, Item 1 at T_1_ and Item 1 at T_2_, Item 2 at T_1_ and Item 2 at T_2_, etc. (Bandalos et al., 1995; Marsh and Yeung, 1997; Guay et al., 1999). Addressing these potential problems, we propose a second iteration (*Iteration 2*), namely: (1) *r*_XT1-STT1_, which depicts the association between the Likert-scale measure X administered at T_1_ and a standardized performance test, denoted as STT1, and (2) *r*_XT2-STT2_, which depicts the association between the same Likert-scale measure X administered at T_2_ and a different standardized performance test, denoted as STT2. Again, in terms of consistency and accuracy, we would expect a similar rating score for the Likert-scale measure X at T_1_ and the performance score of the standardized test at T_1_, and for the same Likert-scale measure X at T_2_ and the performance score of the standardized test at T_2_.

Finally, in order to validate the nature of the Optimal Outcome Questionnaire (Phan et al., 2016) and taking into account the possible shortcomings of Likert-scale measures, we consider a third iteration (*Iteration 3*), which emphasizes the potential associations between the Optimal Outcome Questionnaire and standardized testing at T_1_ and T_2_: (1) *r*_RAB-STT1_, which depicts the association between the RAB Subscale and a standardized test administered at T_1_, and (2) *r*_OAB-STT2_, which depicts the association between the OAB Subscale and a standardized test administered at T_2_. Similar to the two previous iterations, in terms of consistency and accuracy, we would expect a similar rating score for the RAB Subscale and the performance score of the standardized test at T_1_, and the OAB Subscale and the performance score of the standardized test at T_2_.

We contend that the three iterations outlined, in their totality, make substantive contributions to the study of measurement and assessment of optimal functioning. From our rationalization, *r*_XT1-RAB_, *r*_RAB-STT1_, and *r*_XT1-STT1_ would provide theoretical understanding of a current level of functioning [i.e., X_(T1)_ ≈ RAB ≈ ST_(T1)_], whereas *r*_XT2-OAB_, *r*_OAB-STT2_, and *r*_XT2-STT2_ would provide theoretical understanding of an optimal level of functioning [i.e., X_(T2)_ ≈ OAB ≈ ST_(T2)_]. The use of any of the three measures alone is somewhat limited, whereas a combination of two or all three measures is more stringent in terms of elucidating the complex nature of optimal functioning. One notable inquiry that has, to date, remained elusive is our theoretical inference and interpretation of Δ [i.e., Δ_(XT2 − XT1)_, Δ_(OAB-RAB)_, and Δ_(STT2-STT1)_]. For example, given a participant’s response to a Likert-scale measure X at T_1_ and T_2_, the Optimal Outcome Questionnaire at T_1_, and a cognitive competence test at T_1_ and T_2_, can we use this information to explain the process of optimization?

### The Quantification of Optimization: A Proposed Index of Optimization?

The preceding discussion pertaining to the assessment and measurement of optimal functioning is insightful for the purpose of our proposition: *the potential “quantitative” measure of the process of optimization*. Referring to our previous mentioning of the three comparable iterations of optimal functioning, we have X_(T1)_, RAB, and ST_(T1)_ as indicators of a current level of functioning, and X_(T2)_, OAB, and ST_(T2)_ as indicators of an optimal level of functioning. Of interest, in this analysis, is whether and/or to what extent the derivative of Δ notation (e.g., Δ_(XT2 − XT1)_) could align with, and/or fit in with out proposed conceptualization (i.e., activation and enactment of an agent → the sub-process of energization → stimulation of buoyancy of psychological attributes; Figure 1). Empirical validation of optimization (O_B_), as an underlying process, *does not* equate to a “predictive effect,” an “enhancement,” and/or a “causal flow.” In other words, at this stage, methodologically and statistically, very little is known about the “quantitative representation” of the process of optimization. A predictive effect, denoted as a beta value (i.e., *β*), may simply inform us of a positive association between an educational, psychological, or psychosocial agent and an adaptive outcome. For example, in a recent longitudinal, nonexperimental study, Phan et al. (2018a,b,c) found that effective functioning exerted a positive effect on school experience (*β* = 0.62, *p* < 0.001) and academic achievement (*β* = 0.30, *p* < 0.001). Likewise, in an earlier research, McCartney et al. (2007) reported the positive effect of high quality child care, as an index of a psychosocial agent, on school readiness (*β* = 0.21, *p* < 0.01).

A complex issue then relates to the “transformation” of an *r* value (i.e., an association) or a *β* value (i.e., a predictive effect) into an “optimizing effect.” What is an “optimizing” effect, and how do we define and/or calculate this optimizing effect? We postulate that an optimizing effect, denoted as “γ,” is derived from three “pathways,” as shown in Figure 1: (1) Path A describes the result of the activation and enactment of psychological, educational, and psychosocial agents, which then results in the process of energization (i.e., AE → E), (2) Path B describes the result of energization, which consequently leads to the stimulation of buoyancy of different psychological attributes (i.e., E → SB), and (3) Path C describes the arousal of an internal state of functioning and its sustained positioning from T_1_ to T_2_ (i.e., SB → AS).

Having identified these specific paths, we need to conceptualize the “intensity” of optimization by assigning a numerical value to each effect (e.g., 0 for minimal optimizing effect to 1 for maximal optimizing effect). The quantification of *γ*, in this instance, would reflect the totality of effects (i.e., the combined effects of Path A, Path B, and Path C). In other words, as a point of summary: *γ* = Path A + Path B + Path C. An important question for us to consider then, is why would *γ* vary in its magnitude? Referring back to our conceptualization of optimization, one notable aspect is the difference between L_1_T_1_ and L_2_T_2_. The Δ_(L_2_T_2_–L_1_T_1_)_, we argue, is likely to vary in accordance with a person’s current level of functioning (L_1_T_1_) *and* his/her subsequent level of optimal functioning (L_2_T_2_). For example, consider mathematics learning for the topic of *Algebraic expressions* with two different scenarios:

Scenario 1.

L_1_T_1_ = knowing how to solve equations with one unknown, *x*: *x* + 8 = 10, evaluate *x*?
  L_2_T_2_ = knowing how to solve quadratic equations with one unknown, *x*: (*x* – 10)^2^ = 20, evaluate *x*?

Scenario 2.

L_1_T_1_ = knowing how to solve equations with one unknown, *x*: *x* + 8 = 10, evaluate *x*?
  L_2_T_2_ = knowing how to solve simultaneous equations with two unknowns, *x* and *y*: (2*x* + *y*) = 9 and (5*x* – 10*y*) = 20, solve for *x* and *y*.

An analysis of the two mentioned scenarios indicates that L_2_ cognitive functioning is more complex for Scenario 2 (i.e., simultaneous equations that have two unknowns) than that for Scenario 1 (i.e., equations that have one unknown), suggesting that Δ_(L_2_T_2_–L_1_T_1_)_ is “larger” in scale or amount for the former. Achieving L_2_ (i.e., an optimal level) from L_1_ for Scenario 2 requires “more” effort in terms of optimization. This example, interestingly, emphasizes the potential interrelations between the magnitude (i.e., intensity or strength) of the process of optimization and the range or difference between L_1_T_1_ and L_2_T_2_. On this basis, the magnitude of the quantification of *γ* (i.e., reflecting the totality of the process of optimization) is postulated to associate with the “complexity” of L_2_, and how this optimal level of functioning differs from L_1_. In formulating a quantitative derivative of this consideration, we recently proposed a theoretical concept, which we coined as the “index of optimization” (i.e., denoted as IO) (Phan et al., 2019a,b). The IO is defined as: Δ_(L_2_T_2_–L_1_T_1_)_ × *γ*, where *γ* = Path A + Path B + Path C.

The index of optimization is the *combination* (i.e., multiplication) of the difference between L_1_T_1_ and L_2_T_2_ and the magnitude of the optimizing effect of an educational, psychological, or psychosocial agent. How does the IO help us in our understanding of optimal functioning and optimization? A quantified numerical value of IO, which we propose to range from 0 (e.g., minimal IO) to 1, may elucidate the complexity of Δ_(L_2_T_2_–L_1_T_1_)_, and the amount of resources that would be needed for optimization to achieve L_2_ (e.g., ability to solve simultaneous equations with two unknowns)? Importantly, the quantification of IO (close to 1) may also reveal a person’s energy level. A high value of IO, for example, would indicate a person is completely energized, and that the stimulation of buoyancy of different psychological attributes is more likely. A low value of IO (close to 0), in contrast, would indicate a low level of vitality and liveliness.

How do we standardize the measurement and assessment of the IO? Aside from the complexity of Δ_(L_2_T_2_–L_1_T_1_)_, it is important to highlight that the “combination” in effects of Path A (i.e., AE → E), Path B (i.e., E → SB), and Path C (i.e., SB → AS) in the process of optimization is not easily measured and/or computed. Consider the personal experience of energization, which arises from the activation and enactment of educational, psychological, and psychosocial agents. Measurement and assessment of the sub-process of energization, along with the delving into the subsequent arousal and sustaining of an internal state of functioning is a difficult feat to ascertain. It would be of interest for future research to focus on the development of appropriate methodological designs and measurements that could validate and standardize the proposed IO. For example, the level of optimization to assist a person’s optimal level of emotional functioning (e.g., a positive state of happiness) would differ from that of the level of optimization to facilitate optimal physical functioning (e.g., being able to score 50 goals in one football season). A *γ* value of “0.4” for the achievement of optimal cognitive functioning would not, in our view, equate to the same *γ* value of 0.4 for the achievement of optimal physical functioning. In other words, from this comparison, we contend that the index of optimization would vary in accordance with a particular type of functioning (e.g., cognitive functioning *versus* physical functioning).

## Differential Influences of Human Agencies: An Example of Optimal Cognitive Functioning

One notable component of our conceptualization of optimization that is worthy for discussion is the activation and enactment of different agencies to serve as sources of energy. We argue that the differential influences of psychological, educational, and psychosocial agencies are subject to the contextual situation at hand, as well as the timely opportunity that may arise. For example, the optimization of physical functioning (e.g., a football player’s scoring of goals) may benefit more from psychological (e.g., the use of self-efficacy beliefs to convince the football player’s resolve) and/or psychosocial (e.g., the provision of an adequate environment for training) agencies. However, educational agencies (e.g., the teaching of an effective instructional design) could be more appropriate in the optimization of cognitive functioning (e.g., a student’s academic performance in mathematics). In a similar vein, we argue that on a daily basis, the provision of opportunities for optimization purposes may vary in accordance with the contextual situation. What this means is that at any point in time, there are variations in the exposure of psychological, educational, and psychosocial agencies.

Personal energy, we postulate, differentially influence intrinsic motivation, personal resolve, effective functioning, mental strength, and effort expenditure. The stimulation of buoyancy of the five personal attributes that serve to arouse and sustain a person’s progress is likely to vary in accordance with the contextual subject matter. For example, in the context of academic learning, a student may show personal resolve as he or she seeks achievement of optimal best (e.g., achieving mastery of a particular concept). Likewise, an academic subject matter that is of interest and has authentic relevance may energize a student’s intrinsic motivation. In a nonacademic sense, in contrast, an athletic may exhibit a high level of mental strength as he makes attempts to achieve optimal best in long-distance running. The impact of a psychosocial agency (e.g., the provision of emotional and social support) may, in contrast, serve to energize the person’s effort expenditure as she seeks to adjust to a new social environment.

Hence, from our conceptualization, the process of optimization is dynamic in terms of the availability of different agencies. The dynamic of the process of optimization is postulated to intricately link with the contextual matter or situation, at hand – for example, a senior citizen’s seeking to achieve optimal health after surgery, or a student’s fulfillment of mastery competence in Calculus. The contextual matter or situation, from our point of view, then corresponds with a related agency for the personal experience of energization. This consideration places an emphasis on different “pathways” of optimization: (1) psychological agency (e.g., the impact of personal self-efficacy: Bandura, 1997) → energization → stimulation of buoyancy of intrinsic motivation, or (2) educational agency (e.g., an appropriate instructional design: Ngu and Yeung, 2013) → energization → stimulation of buoyancy of effort expenditure, or (3) psychosocial agency (e.g., the impact of teacher-student relationship: Roorda et al., 2011) → energization → stimulation of buoyancy of mental strength.

For this final section of the article, we discuss the comparable influences of psychological, educational, and psychosocial agencies on the optimization of cognitive functioning. From previous research development, we consider the importance of *personal self-efficacy* (Rosenberg, 1965; Bandura, 1997; Trautwein et al., 2006), effective *instructional designs* (Ngu et al., 2014; Star et al., 2015), and *social relationships at school* (Cornelius-White, 2007; Roorda et al., 2011) as psychological, educational, and psychosocial agencies, respectively. Optimal cognitive functioning, within the contexts of schooling, may consist of a student’s academic performance in a subject area, his or her willingness to show mastery competence in a topical theme, or successful school adjustment.

### An Example of Psychological Agency: The Impact of Personal Self-Efficacy

Personal self-efficacy (Bandura, 1997), which forms part of *the self-beliefs system*, is a notable construct that serves as a strong predictor of educational and noneducational outcomes. Personal self-efficacy, according to Bandura (1997), refers to “beliefs in one’s capabilities to organize and execute the course of action required to produce given attainments” (p. 3). This definition contends that self-efficacy is not concerned with a person’s actual capability, but rather his/her self-judgment of perceived competence (e.g., regardless of my current ability, do I believe that I have the capability to complete this mathematics task?) Self-efficacy is a potent predictor of different types of adaptive outcomes (e.g., academic performance), as it mobilizes a person’s state of persistence and effort expenditure, governs his or her choices in life, and regulates appropriate emotional responses. In accordance with Bandura’s (1997) theory, a high level of academic self-efficacy is likely to assist a student to choose an appropriate course of action (e.g., choosing a mathematics-related career pathway: Betz and Hackett, 1983, 1986).

We contend that personal self-efficacy is analogously related to the paradigm of positive psychology (Seligman and Csikszentmihalyi, 2000; Seligman, 2010). As existing research has shown, a heightened state of self-efficacy is associated with improvement in corresponding outcomes (Schunk, 1995; Pajares, 1996a,b;Bandura, 1997). A weakened state of self-efficacy, in contrast, is more likely to result in engagement of maladaptive functioning (e.g., orientation toward performance-avoidance goals: Liem et al., 2008). From the perspective of schooling, in terms of optimization of enjoyable learning experiences, we could use academic self-efficacy as a source of energy to stimulate the buoyancy of intrinsic motivation, personal resolve, effective functioning, mental strength, and/or effort expenditure. To our knowledge, to date, no research has yet considered the conceptualization of academic self-efficacy as an operator of a person’s energy that manifests in his or her stamina and liveliness.

How does academic self-efficacy instill a level of stamina and liveliness in the teaching and learning processes? Our conceptualization, in this case, considers the “potency” of academic self-efficacy to not only predict different types of future educational outcomes (e.g., Fast et al., 2010; Martin et al., 2010; Yailagh et al., 2013), but to also yield a corresponding level of “energy” (i.e., self-efficacy → level of energy). In this analysis, from the characteristics and nature of self-efficacy (Bandura, 1997), we propose that a high level of perceived competence would instill confidence, “feel-good” experiences, and a state of deliberate focus, all of which then transform into a source of energy, acting as an intermediary outcome to stimulate the buoyancy of intrinsic motivation, personal resolve, effective functioning, mental strength, and effort expenditure (i.e., energy → intrinsic motivation, etc.). This postulation of optimization gives a noteworthy positioning of academic self-efficacy (Bandura, 1997) as a source of energy for further prediction.

The proposition regarding a person’s experience of energy, which results from a heightened level of self-efficacy, is an interesting tenet and requires further consideration and development. The main emphasis, in this case, is the saliency of an “interjection” of energy between self-efficacy and a corresponding criterial outcome (i.e., self-efficacy → energy → outcome). Previous correlational studies, in contrast, have attested to the interjecting role of other educational and/or psychological variables. For example, in one of their studies, Pajares and Johnson (1996) used path analysis techniques to highlight the “in-between” role of apprehension between self-efficacy and academic performance. Statistically, taking into account Baron and Kenny’s (1986) writing, it is also appropriate for us to infer that energy could serve as a mediator between self-efficacy and different types of educational outcomes. In the context of optimization, we contend that energy, as an in-between variable, would mediate the effect of academic self-efficacy on intrinsic motivation, personal resolve, effective functioning, mental strength, and/or effort expenditure. An important focus of inquiry, in this case, considers the specific pathways that originate from self-efficacy to intrinsic motivation, personal resolve, effective functioning, mental strength, and effort expenditure, *via* a level of energy. We purport that the stimulation of buoyancy of the five mentioned attributes and their subsequent effects to arouse and sustain progress would vary in accordance with a student’s experience, and the contextual nature of the subject matter. In other words, from this theoretical account, personal experience of energy may selectively influence some but not all of the five attributes. For example, a topical theme that is of interest is more likely to yield a student’s experience of energy that gears toward intrinsic motivation and effort expenditure, whereas another student’s previous experience of repeated successes in a subject matter could energize a high level of personal resolve, effective functioning, and mental strength.

### An Example of an Educational Agency: An Instructional Design

Cognitive load theory (Sweller et al., 2011; Sweller, 2012), for example, has assisted the design and implementation of different instructional designs for effective mathematics learning (e.g., Ngu et al., 2016; Ngu and Phan, 2016). Situating within our explanatory account of optimization, we argue that an instructional design may optimize a student’s mathematics learning experience (e.g., better comprehension and understanding of instructional materials). We consider cognitive load theory as a basis to determine to what extent an instructional design could act as a source of energy during the process of optimization. By this account, a question then is how an instructional design could cultivate positive emotions, which in turn energize a student and stimulates the buoyancy of intrinsic motivation, effective functioning, personal resolve, mental strength, and effort expenditure.

#### Cognitive Load Theory and Element Interactivity

Cognitive load theory (Sweller et al., 2011; Sweller, 2012) highlights the interaction between the acquisition of schemas and a person’s human cognitive architecture. Basically, it focuses on the management of the limited working memory load to process complex cognitive tasks in order to facilitate acquire acquisition. It also seeks to capitalize on the unlimited capacity of the long-term memory that stores a huge number of schemas. Processing schemas retrieved from the long-term memory reduces working memory load.

Sweller (2010) argued that element interactivity is a common factor across the three types of cognitive loads (i.e., extraneous cognitive load, intrinsic cognitive load and germane cognitive load). Element interactivity refers to the interaction between elements within a learning task, which must be processed simultaneously in working memory to allow understanding to occur. An element refers to any item that requires learning (e.g., a number, a symbol, a concept, a procedure, etc.) (Chen et al., 2017). Investing cognitive resources to process interacting elements that hampers learning constitutes extraneous cognitive load, which can be reduced by altering the *design of the instruction.* Investing cognitive resources to process element interactivity that arises from the inherent complexity of material constitutes intrinsic cognitive load. There is an inverse relation between the amount of intrinsic cognitive load and learners’ expertise in a domain. The intrinsic cognitive load of the material is fixed with a given level of the learner’s expertise in the domain. Investing cognitive resources to process element interactivity of the material that contributes toward learning constitutes germane cognitive load. The germane cognitive load depends on the intrinsic cognitive load because the level of element interactivity that determines germane cognitive load is associated with the intrinsic cognitive load of the material.

#### Instructional Design, Cognitive Load, and Emotion

Research has indicated that negative emotions (e.g., anxiety) increase cognitive load imposition and decrease working memory capacity for processing information, resulting in reduced learning (Fraser et al., 2014). However, less is known about the relation between cognitive load imposition, positive emotions, and learning outcomes (Fraser et al., 2012). It is possible an effective instruction that imposes low cognitive load would cultivate positive emotions, which in turn increase a student’s energy level. Based on cognitive load theory, we propose the benefit of acquiring a higher level schema by building on a lower level of schema in learning linear equations.

#### Element Interactivity and Instructional Design

The concept of element interactivity (Sweller, 2010) may provide information that could help us understand the relation between the varying levels of schemas (e.g., lower level schema *versus* higher level schema). According to Sweller (2010), element interactivity acts as an index of complexity of learning material – in other words, the extent to which elements within the learning material interact determines the level of element interactivity. Estimation of the level of element interactivity is made by noting the number of elements involved, as well as assessing the interaction between the elements. Interestingly, in terms of instructional designs, a level of element interactivity accounts for the efficiency of a particular design – for example, a high level of element interactivity imposes high cognitive load and, likewise, vice versa.

**Figure 3:**
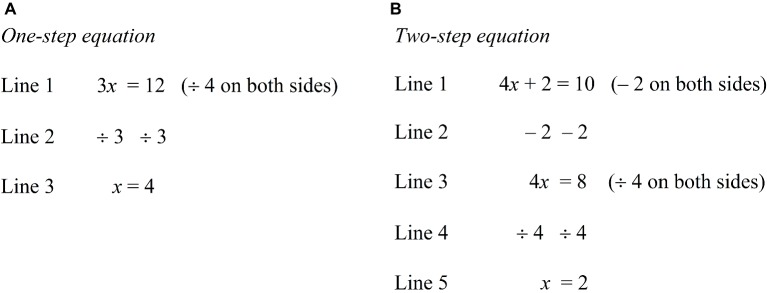
**(A)** One-step equation. **(B)** Two-step equation.

Researchers (Blayney et al., 2009; Ngu et al., 2014) have advocated sequencing complex materials to allow the building of a higher level schema upon a lower level schema (i.e., prior knowledge). In relation to linear equations, capitalizing prior knowledge of one-step equations (Figure 3A) in order to learn two-step equations (Figure 3B) would help ease the burden of the working memory. The learning of the two-step equations can occur in two stages. In the first stage, we can instruct the learner to review the solution procedure of the one-step equation (i.e., Lines 1, 2, and 3, Figure 3A). In the second stage, the learner will learn the solution procedure of the two-step equation (i.e., Lines 1, 2, 3, 4 and 5, Figure 3B). The recall of prior knowledge, in this case, ensures that the learner is able to identify that 3*x* = 12 (i.e., one-step equation) is similar to that of 4*x* = 8 (i.e., Line 3, two-step equation) in terms of problem structure, and therefore both share the same solution procedure. Accordingly, the learning of the two-step equation becomes the learning of Lines 1 and 2 only, thus alleviating working memory load. From this understanding, the acquisition of a higher level schema (i.e., two-step equation) is built upon a lower level schema (i.e., one-step equation), which then reduces the working memory load. Based on the same rationale, we can acquire a higher level schema of a multi-step equation (i.e., 5*x* – 2 = 3*x* + 8) by building on the prior knowledge of a lower level schema of a two-step equation (i.e., 4*x* – 5 = 11).

Our emphasis of the acquisition of a higher level of schema (i.e. complex equations) by building on a lower level of schema (i.e., simple equations) is expected to generate positive emotions, which, in our view, could serve as a source of energy for students. Nonetheless, the prior knowledge level of students may differentially stimulate the buoyancy of the five or a subset of the mentioned attributes in varying degrees of magnitude. In accordance with research in the area of *expertise reversal effect* (Kalyuga et al., 2003), low prior knowledge students need greater instructional support to strengthen their prior knowledge (e.g., one-step equations). Therefore, instructional design that highlights the capitalization of prior knowledge for learning linear equations would have greater impact on the process of optimization for high rather than low prior knowledge students.

It should be noted that popular mathematics textbooks (e.g., Vincent et al., 2012) advocate the learning of linear equations in a hierarchical order of complexity without explicitly indicating the connection between a lower level schema (e.g., one-step equations) and a higher level schema (e.g., two-step equations). This manner of learning linear equations would impose high cognitive load and cultivate negative emotions. Consequently, this would lower the student’s energy level, which is likely to dampen the stimulation of buoyancy of the five mentioned attributes, leading to limited positive arousal and sustainability in the optimization progress.

### An Example of Psychosocial Agency: The Importance of Social Relationships

School is a complex place that may impart conflicting, but yet important information and influences on students, teachers, and school administrators. The school social milieu, in this sense, may influence and shape students’ cognitive, social, moral, and emotional development. This premise places emphasis on the “situational placement” of a student within a larger sociocultural system of change (Okagaki and Sternberg, 1993; Okagaki, 2001). Okagaki’s (2001) proposed *triarchic model of student achievement*, similar to that of Bronfenbrenner’s (1989) *bioecological systems* theory, is interesting as it contends that improvement in cognitive development (e.g., academic performance) is not isolated, but rather depends on extraneous social and educational influences.

Our proposition, described in the preceding sections, considers the school social milieu as a possible agency of optimization. The point of contention is that different individual and/or sociocultural attributes within the social milieu, and not the social milieu itself, would act as optimizing agencies. For example, from Goodenow’s (1993) research, we note that *teachers’ attitudes* toward students and/or *school-based philosophical beliefs* could influence the perceptions of cultural acceptance and diversity, resulting in some students’ negative experiences of school belonging. One notable facet of the school social environment, which could impart meaningful contributions to students’ academic adjustment and learning experiences is that of *teacher-student relationships* (Cornelius-White, 2007; Bergeron et al., 2011; Allen et al., 2013), commonly known as TSRs (Roorda et al., 2011). What is so unique about the concept of TSR as a potential optimizing agency for change?

Roorda et al.’s (2011) theoretical review delves into an interesting tenet, namely, the explanatory account of the concept of TSR in school settings. According to the authors, there are two interesting perspectives that could account and explain the quality of TSRs: *extended attachment* and *social-motivational* perspectives (Note: consult Roorda et al., 2011 for further detail). The extended attachment perspective postulates that teachers, like caregivers, may provide a security base (e.g., emotional security) from which children feel free, and can explore the school environment and engage in different learning and extracurricular activities (Birch and Ladd, 1997; Pianta et al., 1997; Pianta, 1999). Social-motivational perspectives (e.g., self-determination theory: Deci et al., 1991; Ryan and Powelson, 1991), in contrast, contend that children become motivated when they are able to fulfill three fundamental needs: the needs for *relatednes*s, for *competence*, and for *autonomy*. Teachers play a major role, according to Roorda et al. (2011), by showing “involvement (i.e., caring for and expressing interest in the student), providing structure (i.e., setting clear rules and being consequent), and supporting autonomy (i.e., giving students freedom to make their own choices and showing connections between schoolwork and students’ interests)”. Regardless of which theoretical perspective we align to, it is obvious that teachers play a central role in the schooling process.

Teacher-student relationships, consequently, form an important basis at school for social functioning (e.g., Ladd et al., 1999), school adjustment (e.g., Buyse et al., 2009), academic achievement (e.g., Valiente et al., 2008), and engagement in learning activities (Skinner et al., 1990). We expand on this research testament by proposing that a teacher’s role at school could yield a number of meaningful outcomes, which would then transform into a source of energy to differentially stimulate the buoyancy of intrinsic motivation, personal resolve, effective functioning, mental strength, and effort expenditure. In this analysis, from our previous discussion into the operational nature of quality TSRs (Roorda et al., 2011), we consider the importance of the following: (1) a teacher’s persona in-class that conveys messages of warmth, care, and nurturing, (2) a teacher’s attempts to provide opportunities, pathways, and means for student growth, and (3) a teacher’s willingness to facilitate a school social milieu that fosters acceptance, cultural diversity, and a sense of belonging. This development in school, similar to that of self-efficacy and instructional designs, would create a positive learning environment and a strong emotional base for students to learn.

However, the nature of stimulation is subject to different contextual and personal situations. For example, a student’s favorable response to a teacher’s warmth and caring nature may lead to mental strength whereas another student’s response to a teacher’s provision of opportunities and pathways may lead to intrinsic motivation that facilitates effective cognitive functioning and personal resolve. In contrast, a student’s negative experience of school, especially in the relationship with a number of teachers, may result in a low level of mental strength that thwarts learning.

## Conclusion

The study of optimal functioning, which emphasizes the maximization of a person’s capability, requires understanding into the process of optimization. The theoretical concept of optimization has received some research interests, both theoretically and empirically. A synthesis of the literature in the areas of education, psychology, health, and subjective well-being indicates a number of comparable constructs such as cognitive flow, academic buoyancy, and personal thriving. To date, there is no satisfactory account or explanation as to what constitutes optimization. Capitalizing on recent research progress (e.g., Fraillon, 2004; Phan et al., 2017, 2019a), we develop an in-depth account of optimization for further development. We conceptualize optimization as an “underlying process” that could facilitate the achievement of optimal functioning. Optimization, we contend, is more than just a statistical prediction of a psychological variable (e.g., self-efficacy: Bandura, 1997); rather, optimization reflects the experience of “energy,” which then stimulates the buoyancy of intrinsic motivation, effective functioning, personal resolve, mental strength, and effort expenditure.

An important advancement for investigation includes the development of appropriate methodological designs that could test and validate our theoretical contribution of optimization. Our proposed quantification of optimization is useful for assessing a person’s level of optimal functioning, self-referenced against his/her current level of functioning. From our theorization, the index of optimization, quantified as a numerical value, helps us to address specific types of functioning that a person may develop over time (e.g., optimal cognitive functioning *versus* optimal emotional functioning).

In sum, our theoretical contribution into the study of optimal functioning has potential to facilitate specific positive outcomes, academically and nonacademically. The three major optimizing agencies (psychological, educational, and psychosocial) are prevalent as sources of information that enable a person’s experience of energization. What practitioners need to consider are specific pathways and means that could instill and sustain a state of energization to achieve optimal functioning.

Despite the aforementioned theoretical account of optimization, we do acknowledge that more progress is needed to truly understand the nature of optimization. Our proposition (e.g., the concept of “optimizing effect”), as described, is theoretical and conceptual, providing grounding for further empirical development. As Merrotsy (2017) recently noted in his book, titled *Pedagogy for creative problem solving*, there are similar theories such as *flow* (Csíkszentmihályi, 1990, 2014) that lack empirical support – “…It is interesting to note that Thomas (2011) conducted a comprehensive search, using every available database search engine, and was unable to locate any independent research on the existence of flow” (p. 163). This testament, in tandem with our own writing, suggests an important need for researchers to consider pathways and means by which we could soundly “measure, assess, and validate” optimization and, hence, the quantification of flourishing. Importantly too, from this analysis, is a focus on the positive association between optimization and academic performance in school contexts. Our previous description proposes a potential correlation between a child’s experience of optimal best and his/her achievement of a cognitive test (e.g., a quiz in mathematics). It is achievable, in this case, for us to validate this relationship *via* means of factorial and/or regression techniques. What *is* of perplexity, however, is how does the totality of optimization, as detailed in Figure 1, explain a child’s academic performance? At present, we are investigating the operational nature of energy using a quantitative, nonexperimental approach. We encourage readers, likewise, to undertake robust scientific inquiries to support our proposed theoretical-conceptual model of optimization. We are mindful of Merrotsy’s (2017) caution that we do not simply accept and use a theory and/or a concept in a “passing” manner without concrete, established grounding.

## Author Contributions

HP was responsible for the original conceptualization and write-up of the manuscript. BN was responsible for the original conceptualization and write-up of the manuscript. AY assisted in the revision of the manuscript.

### Conflict of Interest Statement

The authors declare that the research was conducted in the absence of any commercial or financial relationships that could be construed as a potential conflict of interest.
